# RUMINA: high-throughput deduplication of unique molecular identifiers for amplicon and whole-genome sequencing with enhanced error correction

**DOI:** 10.1093/bioinformatics/btag097

**Published:** 2026-02-24

**Authors:** Eli Piliper, Stephanie Goya, Alexander L Greninger

**Affiliations:** Department of Laboratory Medicine and Pathology, University of Washington Medical Center, Seattle, 98109, Washington, United States; Department of Laboratory Medicine and Pathology, University of Washington Medical Center, Seattle, 98109, Washington, United States; Department of Laboratory Medicine and Pathology, University of Washington Medical Center, Seattle, 98109, Washington, United States; Vaccine and Infectious Disease Division, Fred Hutchinson Cancer Research Center, Seattle, 98109, Washington, United States

## Abstract

**Motivation:**

Unique molecular identifiers (UMIs) are widely used in next-generation sequencing to enable accurate molecular counting and error correction. However, challenges remain in accurately collapsing UMI clusters, especially when read counts are low or sparse read clusters arise from barcode sequencing errors.

**Results:**

We present RUMINA, a Rust-based pipeline for UMI-aware deduplication and error correction, optimized for both amplicon and shotgun sequencing. RUMINA supports multiple UMI cluster strategies, alongside majority-rule read selection independent of mapping quality, as well as discrete handling of 1–2 read clusters, paired-end merging, and read-length stratification. Benchmarking using simulated HIV population sequencing data and real-world iCLIP and TCR datasets showed that RUMINA improves ultra-low frequency SNV detection (0.01%–1%), reduces false positives, enhances reproducibility, and processes sequencing data up to 10-fold faster than existing tools. By integrating UMI- and sequence-level correction in a high-performance framework, RUMINA offers a fast, scalable, and robust solution for UMI-enabled sequencing workflows.

**Availability and implementation:**

RUMINA is implemented in Rust and distributed as open-source code and precompiled binaries. Source code and installation instructions are available at https://github.com/greninger-lab/rumina. Documentation associated with this manuscript is available at https://github.com/greninger-lab/rumina_paper.

## 1 Introduction

Unique molecular identifiers (UMIs) are short, random nucleotide sequences incorporated during library preparation to uniquely label individual DNA molecules prior to PCR amplification and sequencing. This labeling enables unambiguous read deduplication and correction of sequencing errors by grouping reads originating from the same molecular template (Kivioja *et al.* 2012). UMI-based correction is critical in methodologies requiring detection and quantitation of low-frequency variants such as viral quasispecies analysis (Hauck *et al.* 2018), cancer mutation profiling (Pham *et al.* 2019), single-cell RNA sequencing (Liao *et al.* 2023), and microbial community profiling (Lin *et al.* 2024).

However, challenges remain when UMI clusters contain one or two reads (referred to here as “sparse read clusters” or “singletons” in the software), precluding reliable error correction due to insufficient cluster depth. Such sparse read clusters arise not only from limited sequencing depth but also from errors introduced during UMI synthesis or amplification (Smith *et al.* 2017). These sparse read clusters inflate template counts and reduce correction fidelity (Woerner *et al.* 2021). Current tools such as UMI-tools and UMICollapse perform error correction in the UMIs by clustering UMIs based on sequence similarity and abundance, but none explicitly addresses final sparse read cluster handling. UMI-tools applies abundance and edit distance-based clustering (Smith *et al.* 2017), while UMICollapse employs efficient Burkhard-Keller (BK) tree data structures to cluster UMIs, optimizing speed and memory (Liu 2019). Other pipelines have implemented an approach to handle sparse read clusters, but they were designed and optimized for short amplicon sequencing only (Zhou *et al.* 2021). Neither implements UMI-based error correction of the read sequence.

Shotgun sequencing datasets introduce additional complexity, including variable read lengths and coverage heterogeneity, requiring software capable of scaling to several million reads. Importantly, existing tools rely heavily on mapping quality (MAPQ) scores to select representative reads within UMI clusters. However, MAPQ only reflects alignment confidence relative to a reference genome and may not reflect the most representative read within a UMI cluster, compared to other approaches such as majority voting. Consequently, multiple reads with identical maximum MAPQ scores—some containing sequencing errors—may be arbitrarily chosen, leading to retention of erroneous sequences. This limitation is exacerbated in large datasets with abundant high-MAPQ reads, potentially compromising downstream variant calling and interpretation.

Here, we present RUMINA (Rust-based UMI Networking Analysis), a multithreaded pipeline designed for scalable, accurate UMI error-aware clustering and deduplication compatible with both amplicon and shotgun sequencing. RUMINA integrates discrete sparse read cluster handling and majority-rule consensus collapsing within UMI clusters, overcoming limitations of current MAPQ-based approaches.

## 2 Implementation and algorithms

**Figure 1:**
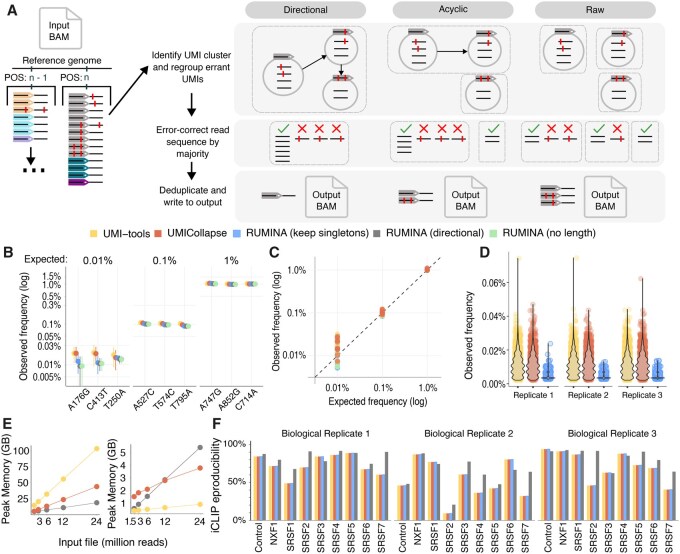
Benchmarking of RUMINA against existing UMI deduplication tools. (A) Overview of RUMINA deduplication workflow and comparison of UMI clustering strategies. In directional and acyclic modes, arrows indicate the merging of UMI clusters based on sequence similarity and abundance; the red bar represents mismatches relative to the consensus sequence. Raw mode merges clusters without any constraints. (B) Detection of simulated HIV variants introduced at frequencies of 1%, 0.1%, and 0.01%; all tools show full recall, but RUMINA reduces SNV frequency overestimation at 0.01%. (C) Correlation between simulated and observed variant frequencies demonstrates improved quantification in RUMINA at ultra-low frequencies. (D) Distribution of false positive variant frequencies, when reported, per tool and method across three replicates. (E) Runtime and memory requirements as a function of input file size. (F) Reproducibility of iCLIP experiments measured as the percentage of peaks shared between biological replicates; RUMINA performs comparably to existing tools.

RUMINA operates on sorted BAM files with UMIs in the read headers, though it can also extract UMIs from FASTQ files before mapping, using user-defined patterns to append them to the headers. RUMINA processes reads coordinate-wise, grouping by exact UMI sequences. Subsequently, RUMINA identifies potential barcode errors using one of the three clustering algorithms (directional, acyclic, or raw), merging low-frequency UMIs within a specified edit distance into high-confidence clusters representing the original molecular template (Fig. 1A). Within each UMI cluster, RUMINA determines the consensus sequence by majority vote. A single representative read, reflecting the dominant sequence, is retained and written to the output BAM file. By default, sparse read clusters (UMIs with one or two reads post-correction) are discarded to minimize noise, although an option (--singletons) enables their retention. Clusters can also be stratified by read length (--length), addressing variable-length reads common in shotgun libraries. The pipeline supports merging of overlapping paired-end reads (--merge-pairs), which avoids incomplete deduplication caused by strand-specific mapping differences.

The three UMI clustering modes are:

1. Directional: an iterative BK-tree-based method adopted from UMI-tools, merging low-frequency UMIs differing by ≤1 edit from a root UMI defined by higher frequency, under a configurable abundance threshold (Smith *et al.* 2017). It assumes sequencing or PCR errors produce low-frequency variants near abundant UMIs.
2. Acyclic: a non-iterative version of Directional mode, restricting merging only to UMIs directly adjacent to the root within edit distance, preventing over-collapsing in datasets with long UMIs or low PCR cycles.
3. Raw: no error correction; UMIs are treated as distinct identifiers.

RUMINA is implemented in Rust for speed and memory safety. Its multithreaded design enables efficient parallelization on large datasets.

## 3 Results

### 3.1 RUMINA enhances ultra-low SNV detection accuracy in simulated HIV data

We evaluated RUMINA’s performance using simulated Illumina paired-end reads derived from an in silico HIV HXB2 population model. Viral populations were generated using a custom Python-based pipeline that introduced single nucleotide substitution variants (SNVs) into independent templates, followed by in silico fragmentation, UMI tagging, deterministic PCR amplification, and Illumina read simulation (Methods, available as supplementary data at *Bioinformatics* online). The dataset comprised 30 000 unique templates containing nine SNVs, generated under predefined constraints to produce three independent variants at each of three allele frequencies (1%, 0.1%, and 0.01%). Only nucleotide substitutions were simulated; insertions and deletions were excluded to isolate UMI-based SNV error-correction performance. PCR and sequencing replicates modeled deterministic amplification dynamics (7 PCR cycles with 0.9 efficiency and 1 × 10^−6^ base error rate) and UMI sequencing errors (10 bp length UMI with 0.5% based on reported Illumina error rates (Stoler and Nekrutenko 2021)). Additional simulation conditions included increased PCR cycles to 10 or 13, UMI length of 8 or 12 nt, and UMI error rates of 0.1% or 1%.

Deduplication was performed using RUMINA (directional, acyclic, and raw modes), UMI-tools, and UMICollapse under settings for paired-end UMI data. Although deduplicated coverage was comparable across methods (average 27 304× for RUMINA versus 32 649× for UMI-tools and 32 650× for UMICollapse), only RUMINA directional and acyclic modes achieved perfect precision (1.0), detecting all true SNVs without false positives (Fig. 1B, Table 1, available as supplementary data at *Bioinformatics* online). The decreased coverage observed with RUMINA was expected due to the removal of singletons and paired-end merging.

While recall was 1.0 for all tools, F1 scores differed substantially, with values of 1.0 for RUMINA, 0.0059 for UMI-tools, and 0.0060 for UMICollapse. UMI-tools and UMICollapse retained numerous singleton UMIs, generating thousands of low-frequency false-positive SNV calls and resulting in near-zero F1 scores. Singleton filtering was not applied to UMI-tools or UMICollapse because these tools do not provide an explicit mechanism to remove singleton UMI clusters during deduplication; consequently, their default outputs retain singleton-derived reads. In contrast, RUMINA removes singleton clusters by default, preventing their contribution to variant calling. When singleton retention was enabled in RUMINA (“keep singletons”), the number of false positive calls increased from zero to 593, and both precision and F1 score similarly collapsed (F1 = 0.029), confirming that singleton-derived errors are the primary driver of the observed performance differences rather than recall.

RUMINA showed accurate frequency estimation across all mutation levels, particularly at 0.01%, where UMI-tools and UMICollapse consistently overestimated allele frequencies (Fig. 1C). RUMINA in default directional mode produced no false positives (Fig. 1D), while the acyclic mode yielded one or two false positives that were not reproduced across replicates and were therefore disregarded (Fig. 1, available as supplementary data at *Bioinformatics* online). In contrast, the raw mode of RUMINA generated eight reproducible false positives (precision = 0.52), and UMI-tools and UMICollapse produced 2995 and 2982 reproducible false positives, respectively, the majority of which were detected at allele frequencies below 0.04% (precision ∼0.003), indicating systematic errors in UMI-based error correction (Fig. 1D).

### 3.2 Impact of UMI parameters and PCR settings on deduplication accuracy

We evaluated the effects of UMI length (8, 10, and 12 nt), PCR cycle number (7, 10, and 13), and UMI substitution error rates (0.1%, 0.5%, and 1%) on deduplication performance. Across all simulated conditions, RUMINA, UMI-tools, and UMICollapse maintained 100% sensitivity for true SNVs. However, UMI-tools and UMICollapse consistently exhibited near-zero F1 scores (∼0.006) across all simulated conditions due to the accumulation of a large number of false-positive calls driven by retained singleton UMIs, as described above (Fig. 2, available as supplementary data at *Bioinformatics* online). RUMINA in directional mode with singleton retention—representing the most comparable configuration to the default strategies of UMI-tools and UMICollapse–achieved higher F1 scores (∼0.03) than both tools despite the low absolute value. This improvement is attributed to RUMINA’s majority-rule representative read selection, which avoids arbitrary read choice in the context of overall high MAPQ scores.

Analysis of UMI cluster structure indicated that 20.7%–22.5% of reads within UMI clusters differed from the dominant read sequence by 1–7 nt, matching simulated sequence error profiles (Table 1, available as supplementary data at *Bioinformatics* online). Cluster size was highly sensitive to both UMI length and UMI error rate. Short UMIs (8 nt) and higher error rates (1%) resulted in extremely large clusters (two and three orders of magnitude larger), suggesting over-merging due to barcode collisions. By contrast, 12 nt UMIs and low error rates (0.1%) produced smaller, well-resolved clusters (average maximum sizes = 257–567 reads), supporting more accurate molecular counting.

### 3.3 RUMINA demonstrates superior runtime and scalability

Benchmarking with input datasets from 1.5 to 24 million reads demonstrated that RUMINA (directional and acyclic modes) consistently outperformed UMI-tools and UMICollapse in processing speed. For instance, RUMINA processed 12 million reads in under 20 minutes using 8 CPU threads, compared to over 100 minutes for UMI-tools. UMICollapse exhibited moderate linear scaling but was still slower (Fig. 1E, Table 2, available as supplementary data at *Bioinformatics* online).

Memory usage remained under 1 GB for UMI-tools, while RUMINA required up to 5.4 GB, and UMICollapse up to 3.8 GB. The increased memory demand is compensated by RUMINA’s significantly faster multithreading execution.

### 3.4 RUMINA improves reproducibility in large-scale iCLIP data deduplication

We applied RUMINA to a large empirical iCLIP dataset encompassing 1.3 billion 1 × 75 bp Illumina reads from 35 FASTQ files across three-to-six biological replicates (Materials, available as supplementary data at *Bioinformatics* online) (Müller-McNicoll *et al.* 2016). This dataset contained 5 nt barcodes at the start of the read sequence (originally incorporated via reverse transcription primers). iCLIP data present challenges due to amplification biases and low UMI diversity, making accurate clustering critical.

RUMINA completed deduplication in 7.7 minutes using 8 threads, outperforming UMICollapse (30.4 minutes) and UMI-tools (78.5 minutes) (Fig. 3, available as supplementary data at *Bioinformatics* online). Memory usage was 2.39 GB for RUMINA, significantly lower than UMICollapse (32.88 GB) but slightly higher than UMI-tools (1.24 GB) (Table 3, available as supplementary data at *Bioinformatics* online). RUMINA maintained consistent performance when run with 1, 4, or 8 threads, demonstrating robust scalability (Tables 3–5, available as supplementary data at *Bioinformatics* online).

Directional UMI clustering with sparse read cluster retention yielded consistent read counts across all tools, confirming clustering algorithm agreement. However, removing sparse read clusters improved biological reproducibility in 77.8% of pairwise comparisons, underscoring the impact of sparse read cluster noise on data quality (Fig. 1F). Sparse read clusters accounted for 8.6%–72.1% of total UMIs, highlighting the importance of their careful consideration in analysis pipelines.

### 3.5 RUMINA resolves clonotype diversity in TCR repertoire sequencing

To assess RUMINA’s impact on immune repertoire profiling, we analyzed UMI-based single-end T-cell receptor (TCR) alpha-chain sequencing data from eight clinical samples (Materials, available as supplementary data at *Bioinformatics* online) (Heather *et al.* 2016). While all tools reported identical deduplicated read counts, the number of detected clonotypes varied, indicating differences in representative read selection (Fig. 4, available as supplementary data at *Bioinformatics* online).

Unlike UMI-tools and UMICollapse, which select the representative reads based on MAPQ scores, RUMINA employs a majority-rule consensus to select the most frequently observed sequence within each UMI cluster. This approach reduces biases introduced by MAPQ tie-breaking heuristics and sequencing errors. In the analyzed dataset, 9.5%–18.0% of UMI clusters contained multiple reads with the highest MAPQ score (Tables 6 and 7, available as supplementary data at *Bioinformatics* online). In such cases, tool-specific deterministic rules were applied to resolve ties, with the chance of overestimating the clonotype diversity.

Consistent with the iCLIP dataset, sparse read cluster UMIs constituted 57.0%–65.2% of clusters, contributing to sequencing depth of coverage but not error correction or reproducibility, highlighting the tradeoff between sensitivity and accuracy.

## 4 Discussion

We present RUMINA, a fast, scalable, and accurate UMI-based deduplication and error correction tool tailored for high-throughput sequencing applications requiring detection of ultra-low (0.01%) frequency variants. Benchmarked against similar established tools (UMI-tools and UMICollapse) on simulated and diverse real datasets (viral heterogeneous population, iCLIP, and immune repertoire sequencing), RUMINA achieves comparable or superior precision and recall while dramatically reducing runtime by up to tenfold.

A key innovation of RUMINA is its majority-rule representative read selection, which bypasses reliance on MAPQ scores prone to ambiguity in UMI clusters with multiple top-scoring reads. This sequence-driven approach minimizes retention of erroneous reads, improving variant calling accuracy and reproducibility.

Sparse read cluster UMIs, prevalent across datasets (up to 72%), remain a challenge due to insufficient cluster depth for error correction. By default, RUMINA filters sparse read clusters to enhance accuracy, though they may be retained to maximize sensitivity depending on experimental goals. Our results in iCLIP data demonstrate that sparse read cluster removal substantially improves reproducibility without compromising overall clustering fidelity.

Our findings emphasize the critical interplay of experimental design parameters (UMI length, PCR cycles, and template diversity) in minimizing barcode collisions and amplification biases. Longer UMIs (≥10 nt) combined with moderate PCR cycles optimize cluster resolution and specificity.

While RUMINA demonstrates strong performance across a range of UMI-based NGS applications, several limitations should be noted. RUMINA selects representative reads by frequency, which may be biased if early PCR or sequencing errors dominate a cluster. Its compatibility with single-cell UMI + cell barcode formats remains untested, limiting current use in scRNA-seq. Simulations were performed on partial HIV sequences due to the computational cost of modeling PCR amplification and error propagation on larger genomes, though real-life data using iCLIP and TCR datasets were also analyzed. RUMINA requires more memory than UMI-tools and UMICollapse, reflecting a tradeoff between speed and memory usage. This is mainly due to in-memory graph construction. While acceptable for most shotgun datasets, future work will explore optimizations such as memory mapping to reduce resource demands.

## Supplementary Material

btag097_Supplementary_Data

## Data Availability

Data associated with this article can be accessed at https://github.com/greninger-lab/rumina_paperand the following zenodo repositories: https://doi.org/10.5281/zenodo.18167358, https://doi.org/10.5281/zenodo.18167426, https://doi.org/10.5281/zenodo.18167490, https://doi.org/10.5281/zenodo.18167533, https://doi.org/10.5281/zenodo.18167542, https://doi.org/10.5281/zenodo.18176728, https://doi.org/10.5281/zenodo.18167580, and https://doi.org/10.5281/zenodo.18176787.
