# Supplementary material for: RUMINA: high-throughput deduplication of unique molecular identifiers for amplicon and whole-genome sequencing with enhanced error correction
Source: Bioinformatics. 2026 Feb 24;42(3):btag097. doi: 10.1093/bioinformatics/btag097 (PMC12975283; doi:10.1093/bioinformatics/btag097)
Supplement: btag097_Supplementary_Data [file btag097_supplementary_data.zip › RUMINA_20250926_Supplementary_Methods_R1_260120_clean.docx]

**Supplementary Methods**

**Benchmarking**

All FASTQ simulation and benchmarking was performed on a 2022 Mac Studio with a 10-core M1 Max CPU and 64 GB of memory, running macOS Sonoma 14.5. Testing of each dataset with all software tools, as well as memory monitoring, was automated with the memtest.py script (https://github.com/greninger-lab/rumina_paper). Below we detail how BAM files were generated; due to their large size, all analyzed BAM files are available upon request.

For all the following analyses we used RUMINA v0.9.81, UMI-tools v1.1.6 (Smith *et al.*, 2017), and UMICollapse v1.0.0 (Liu, 2019).

**Datasets and RUMINA software**

The RUMINA software is available for download and installation from the GitHub repository: https://github.com/greninger-lab/rumina

Detailed information on software dependencies and installation procedures is provided in the repository README file.

All code, workflows, and pipelines required to reproduce the benchmarking analyses presented in this study are available in the companion GitHub repository: https://github.com/greninger-lab/rumina_paper

Due to their large size, several datasets used in this study were deposited in Zenodo. These datasets can be accessed at the following links:

https://zenodo.org/records/18176787 (RUMINA - hiv_sim dataset)

https://zenodo.org/records/18167580 (RUMINA - TCR dataset)

https://zenodo.org/records/18167358 (RUMINA - iCLIP datasets - inputs part 1)

https://zenodo.org/records/18167426 (RUMINA - iCLIP datasets - inputs part 2)

https://zenodo.org/records/18167490 (RUMINA - iCLIP datasets - analysis/outputs part 1)

https://zenodo.org/records/18167533 (RUMINA - iCLIP datasets - analysis/outputs part 2)

https://zenodo.org/records/18167542 (RUMINA - iCLIP datasets - analysis/outputs part 3)

https://zenodo.org/records/18176728 (RUMINA - iCLIP datasets - analysis/outputs part 4)

**Cluster composition analysis**

To view the composition of read clusters before deduplication, we used RUMINA’s --only-group command to add a UG tag to each output read identifying its assigned UMI cluster. This mode only outputs reads without deduplicating them. Output reads were written to a BAM file subsequently sorted by the UG tag and processed with our *tag_splitter* CLI tool (<https://github.com/greninger-lab/rumina_paper>), which reported various metrics on cluster composition such as read count, number of reads differing from majority sequence, and other details. These steps were achieved with the generalized Bash code below:

rumina -t 2 <FILE> -g directional --singletons --only-group –outdir <OUTDIR> -s _ &&

samtools sort -@ 10 -t UG <OUTDIR>/<OUTPUT FILE> -o <OUTDIR>/<OUTPUT_FILE_PREFIX>_tagsorted.bam

tag_splitter -i <OUTDIR>/<OUTPUT_FILE_PREFIX>_tagsorted.bam -d <TEMP_DIR> -t UG -o <OUTPUT_FILE_PREFIX>_cluster_report.tsv.

**Simulation of viral populations with known low-frequency variants**

To benchmark UMI deduplication in whole genome sequencing data under controlled conditions, we simulated a heterogeneous HIV-1 population with known single nucleotide variants (SNVs) at predefined frequencies. Custom pipeline scripts were executed using Python 3.13.7 with Biopython 1.85. Amplicon sequencing reads were simulated using ART-Illumina v2.5.8 with the HS25 model, generating paired-end 150 bp reads. Parallel execution was performed using GNU Parallel 20250822. All analyses were run on macOS.

Briefly, starting from a FASTA file containing the first 1,000 nt of the HXB2 reference genome (NCBI GenBank accession number K03455.1), we generated 30,000 independent copies of the sequence and introduced nine predefined single-nucleotide substitution variants (SNVs), with three independent variants per frequency tier (1%: C714A, A747G, A852G; 0.1%: A527C, T574C, T795A; and 0.01%: A176G, T250A, C413T). This step was performed with the custom pipeline 1_simulate_population_fasta.py (https://github.com/greninger-lab/rumina_paper/tree/main/hiv_simulation). Insertions and deletions were not simulated. This design ensured balanced representation of ultra-low-frequency substitutions while avoiding linkage between variants within the same template.

From this baseline population, independent simulated datasets were generated for each combination of UMI length (8, 10, or 12 nt), PCR cycle number (7, 10, or 13 cycles), and UMI substitutions error rate (0.1%, 0.5%, or 1%). For each dataset, templates were fragmented in silico (mean fragment length = 300 bp, SD = 100 bp), tagged with synthetic UMIs in the FASTA header, and amplified *in silico* using a deterministic PCR model with 0.9 amplification efficiency. PCR jackpotting or stochastic effects were not modeled. These steps were performed using the custom pipeline 2_simulate_fragmentation_and_pcr.py (https://github.com/greninger-lab/rumina_paper/tree/main/hiv_simulation).

To account for PCR saturation effects and to constrain computational load, a maximum copy-number cap was applied that varies with the number of PCR cycles, thereby capturing the plateau phase of PCR. For 7 cycles, a fixed cap of 300 copies was used, representing slightly more than three times the theoretical expected copy number ((1+0.9)^7^= 89 copies). For 10 and 13 cycles, a more conservative saturation factor of 2 was employed to maintain computational efficiency, capping the maximum copies at twice the expected values (1200 and 8500 copies, respectively). To incorporate experimental variability, the number of PCR copies per template was drawn from a normal distribution centered on the expected copy number with a 10% standard deviation. A per-nucleotide substitution error rate of 1×10⁻⁶ was applied to each PCR copy, simulating polymerase-induced replication errors during amplification. Errors were introduced as random base substitutions independently at each position, without accumulation across cycles. To standardize computational requirements across different experimental conditions, simulated amplicon datasets derived from the 10-cycle and 13-cycle PCR amplifications were downsampled to a maximum of 6 million sequences using BBMap reformat (https://sourceforge.net/projects/bbmap/). This artificial subsampling also introduced UMI singletons, mimicking realistic sequencing scenarios where low-abundance molecules may be observed only once due to limited sampling depth.

Finally, Illumina paired-end reads (2x150 bp) were simulated from the resulting amplicons using ART with the -amp option enabled, which prevents sequence fragmentation with the custom pipeline 3_art_illumina_chunks.sh (https://github.com/greninger-lab/rumina_paper/tree/main/hiv_simulation). The Illumina HiSeq 2500 sequencing profile (HS25 model) was used, with 1× coverage per amplicon generated during PCR simulation (Huang *et al.*, 2012). All reads retained UMI information in their FASTQ headers. UMI barcodes were subject to simulated substitutions errors at three different rates 0.1%, 0.5% or 1% in amplicon reads, except for those FASTA sequences derived from original sequence templates, which retained error-free UMI sequences.

After FASTQ simulation under different conditions were created, they were mapped against the original sequence HIVB HXB2 used to generate the files templates using BWA MEM v0.7.19. Sorted BAM files were deduplicated with RUMINA using paired end merging and stratifying by read length, with the three clustering methods (directional, acyclic and raw). RUMINA was run by default, therefore removing singletons, however this feature only worked on the 10 and 13 PCR simulation as they were downsampled just before FASTQ generation, while the other datasets processed all created in silico molecules. UMI-tools and UMICollapse also were run with the best parameters possible for this dataset (directional method with paired end mode).

*UMI-tools:*

umi_tools dedup -I <FILE> --umi-separator : --method directional --stdout <OUTFILE> --random-seed 0 --paired

*UMICollapse:*

umicollapse bam -i <FILE> --umi-sep : -o <OUTFILE> --paired

*RUMINA, the three clustering methods, stratified by length:*

rumina <FILE> --grouping_method directional --separator : --threads 8 --paired –length

rumina <FILE> --grouping_method acyclic --separator : --threads 8 --paired --length

rumina <FILE> --grouping_method raw --separator : --threads 8 --paired --length

Deduplicated and sorted BAM files were analyzed using iVar v1.4.4 to evaluate the number of true positives (TP) and false positives (FP) among low- and ultra-low-frequency variants (Grubaugh *et al.*, 2019). To this end, all SNVs with a frequency above 0.001% and a minimum depth of 3X were called. The SNV calls were analyzed in RStudio (R version 4.2.2), focusing on nucleotide substitutions and disregarding the Fisher’s exact test implemented in iVar as a filtering parameter, since the test was originally designed for non-UMI-based sequencing strategies and may not be appropriate for deduplicated UMI data. The complete results, including plots and statistical analyses for the comparative study of low-frequency SNVs, are available in the RMarkdown document at: https://github.com/greninger-lab/rumina_paper/tree/main/hiv_simulation

*iVAR variants:*

samtools mpileup -aa -A -d 0 -B -Q 0 --reference <hiv ref> $input | ivar variants -p $outfile -t 0.00001 -m 3 -r <hiv ref>

**iCLIP dataset**

FASTQ files of individual-nucleotide resolution crosslinking and immunoprecipitation (iCLIP) sequencing targeting the RNA-binding protein SRSF were downloaded from NCBI BioProject accession number PRJNA286202 (Müller-McNicoll *et al.*, 2016; Smith *et al.*, 2017). Briefly, the authors identified endogenous RNA targets of seven GFP-tagged SR proteins and NXF1 using transcriptome-wide in Mus musculus P19 cells using iCLIP with UV crosslinking at 254 nm and anti-GFP immunoprecipitation. Recovered RNA was reverse transcribed with primers containing 5-nt UMI following library prep protocol for Illumina sequencing. Sequencing was performed in 75-nt single-end mode on an Illumina HiSeq2000 platform. For our benchmarking, we processed the published FASTQ files and mapped them to the mm9 reference genome (NCBI GenBank accession number CM000994.1), as performed in the original publication (Müller-McNicoll *et al.*, 2016). Sorted BAM were deduplicated using RUMINA, UMI-tools and UMICollapse with the directional method. iCLIP reproducibility was calculated as the proportion of cross-linked nucleotides with depth ≥ 2X in one replicate also present in another replicate (König *et al.*, 2010) (https://github.com/greninger-lab/rumina_paper/tree/main/iclip). Three replicates per sample (27 total) were used to match the prior UMI-tools analysis (Smith *et al.*, 2017).

*UMI-tools:*

umi_tools dedup -I <FILE> --umi-separator _ --method directional --stdout <OUTFILE> --random-seed 0

*UMICollapse:*

umicollapse bam -i <FILE> --umi-sep _ -o OUTFILE

*RUMINA for amplicon sequencing:*

rumina <FILE> --grouping_method directional --separator _ --threads 8

*RUMINA for amplicon sequencing keeping singletons:*

rumina <FILE> --grouping_method directional --separator _ --singletons --threads 8

**T Cell Receptor sequence dataset**

FASTQ files from T cell receptor (TCR) repertoire UMI-based amplicon sequencing from eight healthy individuals were downloaded from NCBI SRA (accession number SRR1543964- SRR1543971). Briefly, the authors amplified by PCR the TCR V(D)J rearrangements from mRNA using reverse transcription primers directed against the constant region and 5'-ligation-RACE, incorporating a twelve-nucleotide random nucleotide barcode (UMI) prior to amplification (Heather *et al.*, 2016). Sequencing was performed in single-end mode on an Illumina MiSeq platform. For our benchmarking, we process the FASTQ files were and mapped against human genome hg38 GCA_000001405.29, following previously described methodology (Mitchell *et al.*, 2020). Sorted BAM files were deduplicated in triplicate using RUMINA, UMI-tools, and UMICollapse with the directional method (https://github.com/greninger-lab/rumina_paper/tree/main/tcr). RUMINA was run in two modes: removing singletons (default) and preserving singletons, to compare output with the other tools. TCR assemblies and CDR3 sequences were reconstructed using TRUST4 v1.1.5 with default parameters to assess clonal diversity and “out of frame” CDR3aa sequences were filtered out from the output before further analysis (Song *et al.*, 2021).

Using our *tag_splitter* tool (see Cluster composition analysis), we observed that maximum observed deviance from majority sequence (in bases) across clusters was high (89bp with a 238bp read length) in this dataset. To explore this apparent cluster heterogeneity further, we first defined highly discrepant reads (relative to sequence majority) as having at least 25% of read length (60/238 bases) differ from majority sequence. We then measured the maximum number of discrepant positions across reads per cluster with at least one discrepant read and calculated the mean (6.26) and SD (6.43) of this metric. We defined the threshold for highly discrepant clusters as outside 9 standard deviations of the mean, just above the 60 base / 25% threshold. We found 33 clusters (0.0074% of the entire dataset and 0.027% of sequence-heterogeneous clusters) to be highly discrepant. High-difference reads in these clusters were generally the extreme minority, presented with a lower mapping quality, and were likely QC-fail reads that could not be filtered due to a lack of Phred score data in the dataset.

*UMI-tools:*

umi_tools dedup -I <FILE> --umi-separator _ --method directional --stdout <OUTFILE> --random-seed 0

*UMICollapse:*

umicollapse bam -i <FILE> --umi-sep _ -o OUTFILE

*RUMINA for amplicon sequencing:*

rumina <FILE> --grouping_method directional --separator _ --threads 8

*RUMINA for amplicon sequencing keeping singletons:*

rumina <FILE> --grouping_method directional --separator _ --singletons --threads 8

*TRUST4:*

run-trust4 -b $bam -f hum_bcrtcr.fa --ref human_IMGT+C.fa -t 10 -o $outfile

**References**

Grubaugh,N.D. *et al.* (2019) An amplicon-based sequencing framework for accurately measuring intrahost virus diversity using PrimalSeq and iVar. *Genome Biol.*, **20**, 8.

Heather,J.M. *et al.* (2016) Dynamic Perturbations of the T-Cell Receptor Repertoire in Chronic HIV Infection and following Antiretroviral Therapy. *Front. Immunol.*, **6**.

Huang,W. *et al.* (2012) ART: a next-generation sequencing read simulator. *Bioinformatics*, **28**, 593–594.

König,J. *et al.* (2010) iCLIP reveals the function of hnRNP particles in splicing at individual nucleotide resolution. *Nat. Struct. Mol. Biol.*, **17**, 909–915.

Liu,D. (2019) Algorithms for efficiently collapsing reads with Unique Molecular Identifiers. *PeerJ*, **7**, e8275.

Mitchell,K. *et al.* (2020) Benchmarking of computational error-correction methods for next-generation sequencing data. *Genome Biol.*, **21**, 71.

Müller-McNicoll,M. *et al.* (2016) SR proteins are NXF1 adaptors that link alternative RNA processing to mRNA export. *Genes Dev.*, **30**, 553–566.

Smith,T. *et al.* (2017) UMI-tools: modeling sequencing errors in Unique Molecular Identifiers to improve quantification accuracy. *Genome Res.*, **27**, 491–499.

Song,L. *et al.* (2021) TRUST4: immune repertoire reconstruction from bulk and single-cell RNA-seq data. *Nat. Methods*, **18**, 627–630.
