# Supplementary material for: RUMINA: high-throughput deduplication of unique molecular identifiers for amplicon and whole-genome sequencing with enhanced error correction
Source: Bioinformatics. 2026 Feb 24;42(3):btag097. doi: 10.1093/bioinformatics/btag097 (PMC12975283; doi:10.1093/bioinformatics/btag097)

Supplementary Figure 1. A) Detection of simulated HIV variants introduced at frequencies of 1%, 0.1%, and 0.01%; all tools show full recall, but RUMINA reduces SNV frequency overestimation at 0.01%. C) Correlation between simulated and observed variant frequencies demonstrates improved quantification in RUMINA at ultra-low frequencies. C) Distribution of false positive variant frequencies, when reported, per tool and method across three replicates.

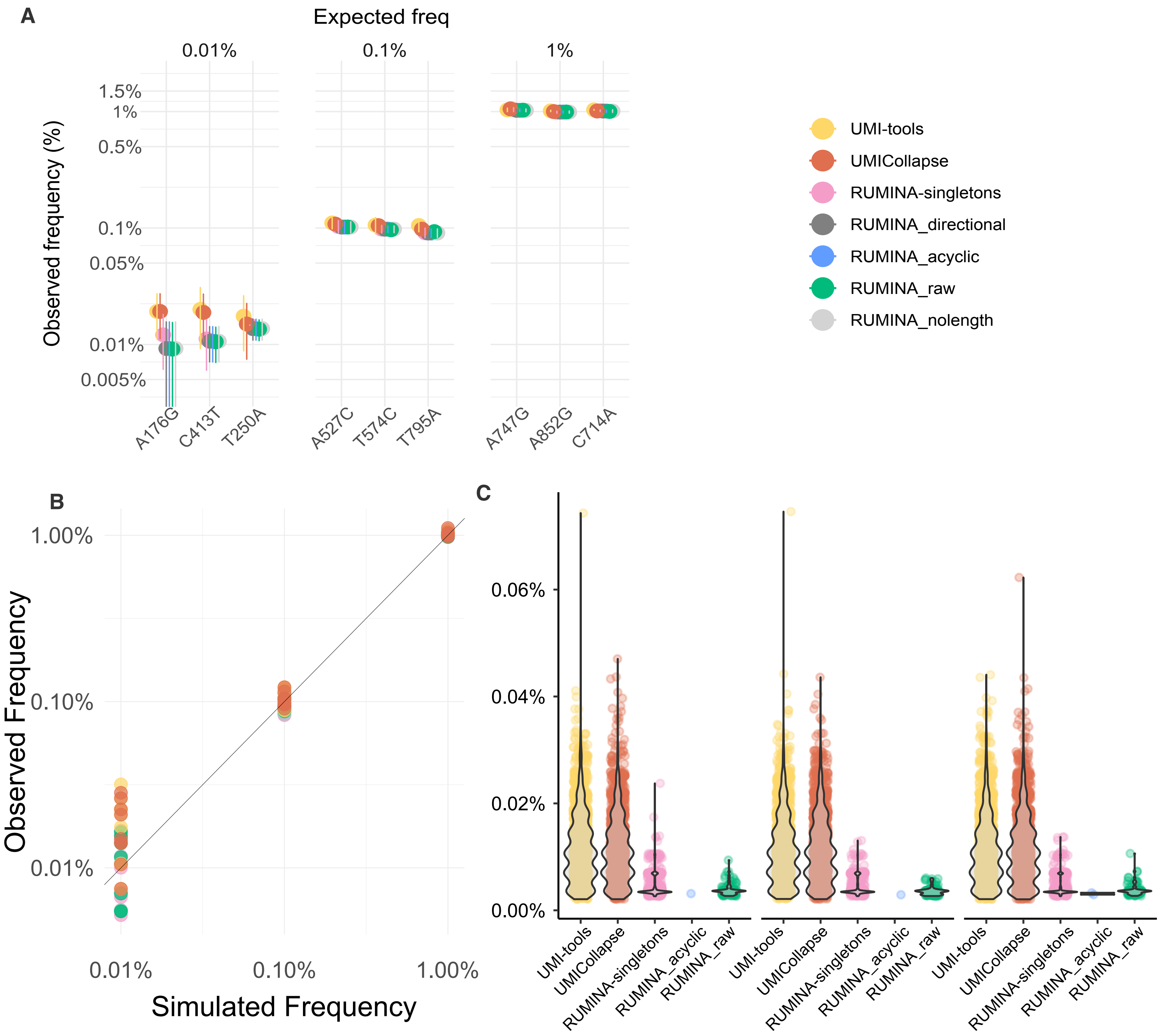

Supplement: btag097_Supplementary_Data [file btag097_supplementary_data.zip › RUMINA_SupplementaryFigure1.pdf]
