# Supplementary material for: RUMINA: high-throughput deduplication of unique molecular identifiers for amplicon and whole-genome sequencing with enhanced error correction
Source: Bioinformatics. 2026 Feb 24;42(3):btag097. doi: 10.1093/bioinformatics/btag097 (PMC12975283; doi:10.1093/bioinformatics/btag097)

Supplementary Figure 3. Memory usage and runtime of RUMINA directional, UMI-tools and UMICollapse tested on 3 iterations for iCLIP and TCR sequencing datasets.

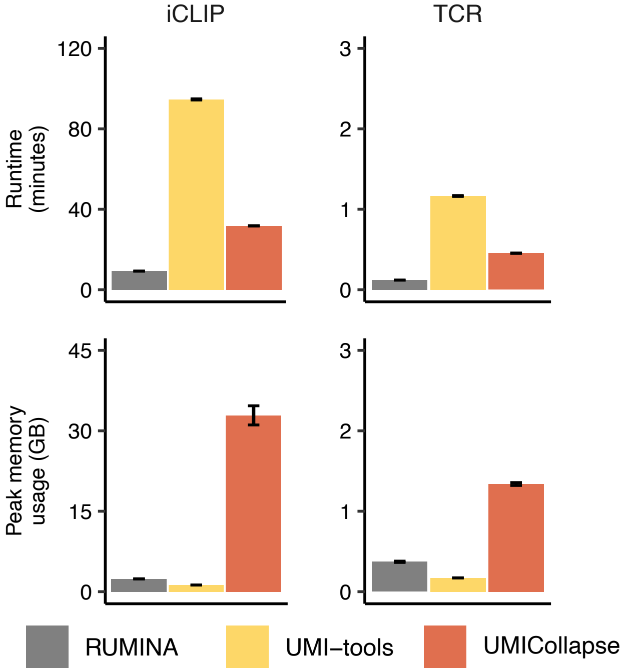

Supplement: btag097_Supplementary_Data [file btag097_supplementary_data.zip › RUMINA_SupplementaryFigure3.pdf]
