# Supplementary material for: RUMINA: high-throughput deduplication of unique molecular identifiers for amplicon and whole-genome sequencing with enhanced error correction
Source: Bioinformatics. 2026 Feb 24;42(3):btag097. doi: 10.1093/bioinformatics/btag097 (PMC12975283; doi:10.1093/bioinformatics/btag097)

Supplementary Figure 4. Percentage of shared and unique TCR clonotypes identified with TRUST4 after UMI deduplication using RUMINA (directional method), UMI-tools, and UMICollapse across all analyzed samples. A) The x-axis represents the percentage of total per-sample clonotypes that are unique to each method or method combination. The two panels differ in how RUMINA handles singleton UMI clusters (clusters with 1 or 2 reads): Left panel: Results when RUMINA retains singletons in the deduplicated output. Right panel: Results when RUMINA removes singletons (default setting). B) Correlation between the number of output reads (equivalent to the number of UMI clusters identified) when using RUMINA with singletons retained, versus UMI-tools or UMICollapse. The complete correlation indicates that the directional method implemented in the three tools yields consistent deduplication results.

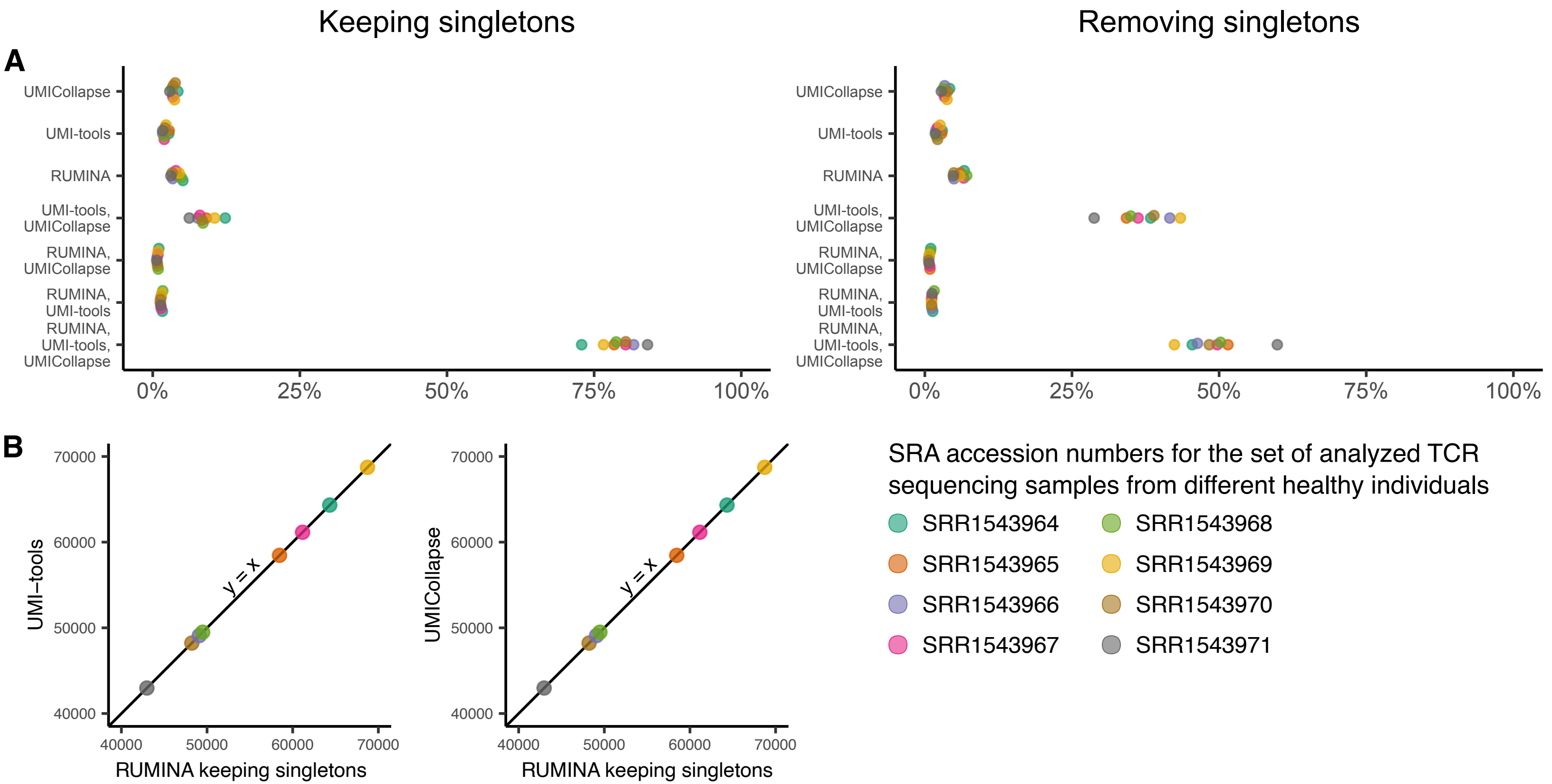

Supplement: btag097_Supplementary_Data [file btag097_supplementary_data.zip › RUMINA_SupplementaryFigure4.pdf]
