# Supplementary material for: RUMINA: high-throughput deduplication of unique molecular identifiers for amplicon and whole-genome sequencing with enhanced error correction
Source: Bioinformatics. 2026 Feb 24;42(3):btag097. doi: 10.1093/bioinformatics/btag097 (PMC12975283; doi:10.1093/bioinformatics/btag097)

Supplementary Figure 2. Mean F1 score and standard deviation across in silico replicates for each UMI deduplication tool under different simulated conditions. Each panel corresponds to one tool (or tool settings), and each point represents the average F1 score across replicates for a given condition, with error bars indicating one standard deviation. Conditions are labeled using the UMI length (8, 10 or 12), UMI sequencing error rate (0.1%, 0.5%, or 1%), and PCR cycle number (7, 10 or 13), separated by underscores.

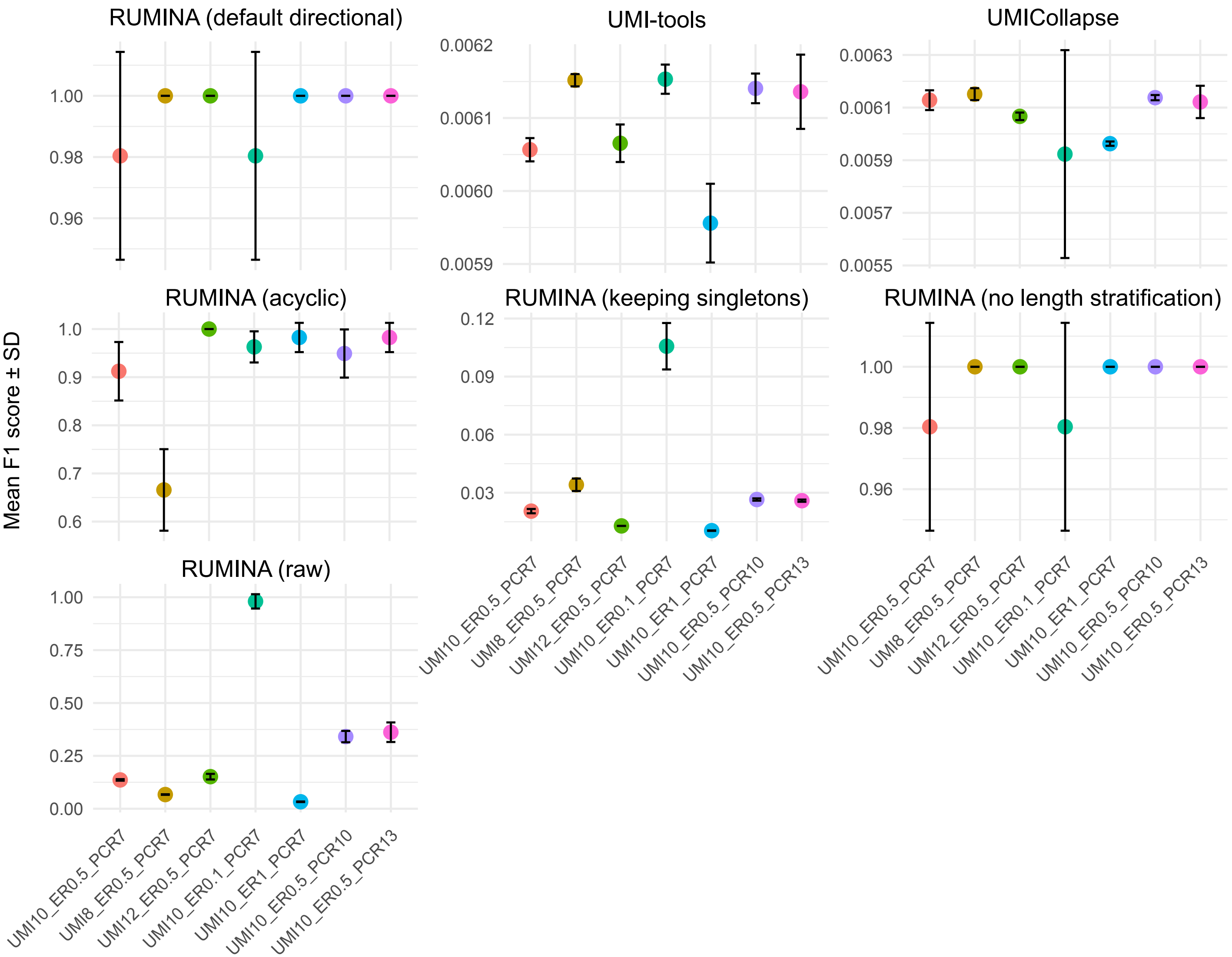

Supplement: btag097_Supplementary_Data [file btag097_supplementary_data.zip › RUMINA_SupplementaryFigure2_R1.pdf]
